# Farmers’ perceptions of navy bean (*Phaseolus vulgaris* L.) production constraints, preferred traits and farming systems and their implications on bean breeding: a case study from South East Lowveld region of Zimbabwe

**DOI:** 10.1186/s13002-021-00442-3

**Published:** 2021-03-12

**Authors:** Bruce Mutari, Julia Sibiya, Eileen Bogweh Nchanji, Kennedy Simango, Edmore Gasura

**Affiliations:** 1 School of Agricultural, Earth and Environmental Sciences, University of KwaZulu-Natal, P. Bag X01, Scottsville, Pietermaritzburg, 3209 South Africa; 2 Department of Research and Specialist Services, Crop Breeding Institute, Harare, Zimbabwe; 3 Alliance Bioversity - International Center for Tropical Agriculture (CIAT), Nairobi, Kenya; 4 Marondera University of Agricultural Sciences and Technology, Marondera, Zimbabwe; 5 University of Zimbabwe, Mt Pleasant, Harare Zimbabwe

**Keywords:** Navy bean, Participatory rural appraisal, Production constraints, Preferred traits, Marketing constraints

## Abstract

**Background:**

Navy bean is an important legume crop in Zimbabwe. Although its production in Zimbabwe is limited by multiple constraints including biotic, abiotic and socio-economic, there is no documented evidence. Thus, this study aimed at identifying farmers’ production constraints, preferred traits and cultivars of navy bean, and strategies used to mitigate some of these constraints.

**Methods:**

A Participatory Rural Appraisal approach involving transect walks, focus group discussions (FGDs), and formal surveys with semi-structured questionnaires was conducted in four villages of the Lowveld region of Zimbabwe. In each of the four villages, two FGDs (one for men and one for women) were conducted. A total of 176 (75 males and 101 females) navy bean-growing households were interviewed. Data from household interviews and FGDs was analysed using the Statistical Package for Social Scientists computer package.

**Results:**

The most important constraints to navy bean production were drought stress (Females—86%, Males—73%), heat stress (Females—58%, Males—55%), power outages (Females—46%, Males—54%), poor soil fertility (Females—32%; Males—33%) and susceptibility to pod shattering (Females—32%, Males—43%). Mitigation strategies included mulching (18%), ridges (12%), reduced acreage (11%), and cultivating to retain more soil moisture (11%) for drought stress, while irrigating at night (32%), and adjusting planting dates (29%) were used to manage heat stress. Farmer-preferred traits included tolerance to drought and heat, early maturing varieties and disease resistance. Marketing constraints included non-payment for produce in hard currency, lack of diversity in terms of off-takers, high inflation, low grain producer price, delayed payment and breach of contract by contractors.

**Conclusion:**

There will be increased adoption of improved navy bean cultivars if breeding programs address the aforementioned constraints and consider farmer-preferred traits when developing new cultivars. Breeders should work closely with extension officers to ensure that cultivars released are cultivated with appropriate agronomic packages for increased productivity and high adoption.

## Background

Globally, common bean (*Phaseolus vulgaris* L.) is an important food and nutritional security pulse crop that provides a cheap source of vegetable proteins, micronutrients (iron and zinc, and vitamins) and dietary fibre [1]. In addition, it serves as an income-generating crop thereby supporting many livelihoods, especially in sub-Saharan Africa (SSA). Common bean is widely cultivated, and the largest volumes of the crop have come from SSA and Latin America, which account for more than 60% of the world production [2, 3]. In southern and eastern Africa, the major producers are mainly smallholder farmers who primarily depend on beans for their livelihoods, household food, nutrition and income security [2, 4]. Different market classes (sugar, red mottled and navy bean) of common bean including landraces are grown by farmers in the Lowveld region of Zimbabwe [5].

Navy beans (dry oval pea-sized white haricot bean), locally called Michigan pea bean, is a distinct cultigen of common bean which is grown for both income and food security. Due to its high market value compared with the other bean market classes, smallholder farmers in the South East (SE) Lowveld region grow it mostly for income generation, almost exclusively for the bean canning industry under contractual agreements with canning companies, including Cairns Foods Limited, Olivine Industries Limited, Africa Preserves, and National Foods. Income from contract farming (contractor provides inputs such as seed, fertilizers and chemicals) is used to cater for household needs, purchase livestock (goats and cattle), pay children’s school fees and for re-investment on the farm and other commercial enterprises [5]. Although this is not documented, information gathered during various field days with navy bean farmers indicates that farmers retain around 10% of the produce for household consumption, and 90% is delivered to the processor for income generation [Personal observation, April 2019]. Even though navy bean is an important food security crop, the high market value associated with the crop forces farmers to deliver a greater percentage of the produce to the processor in search of income. The rapidly expanding urban population in Zimbabwe is driving a greater demand for more processed food products. Moreover, preference for fast cooking foods and off-shelf products such as canned beans is expected to increase in Zimbabwe due to changes in eating habits (associated with urbanization), rising costs of cooking fuels and electricity.

Although navy bean is a major cash crop, its production, productivity and market supply in Zimbabwe has declined in recent years due to several constraints [Mukweza, personal communication, 20 May, 2018^1^]. The challenges include low grain yield, high susceptibility to diseases, unavailability of locally bred improved cultivars, drought and heat stress, damages by field pests including bean stem maggot (*Ophiomia phaseoli* sp.), aphids (*Aphis fabae*) and storage pests (*Acanthoscelides obtectus*) [5]. The unavailability of improved cultivars results in farmers planting old cultivars which are low yielding and susceptible to new races of diseases. On the other hand, insect pests (cause grain damage), diseases (cause grain discolouration), drought and heat stress (reduce grain size) affect the canning quality of the grain resulting in the rejection of the commodity by food processors. If a commodity is rejected by a food processor due to the above-mentioned constraints, the farmer would have lost income. This makes it difficult for a farmer to plant the next subsequent crop due to lack of running capital or income. Consequently, navy bean farmers have accumulated indigenous knowledge and experience over time on production systems and how to cope with a wide range of biotic, abiotic and socio-economic constraints. It is, therefore, important to utilize this knowledge from the farmers during cultivar development to improve the adoption rate of newly developed navy bean cultivars.

Due to the low productivity and inadequate grain supplies of navy beans, local supplies meet about 40% of the national requirement [6]. Therefore, bean processors in Zimbabwe are obliged to import navy bean grain (about 60%) from countries such as Ethiopia, South Africa, China, Zambia and Malawi [6]. On monthly basis, canning companies in Zimbabwe import more than 100 MT of navy bean grain worth around USD124,000 [6]. It is, therefore, important that the local navy bean production be increased to meet the required quantities. Significant navy bean production in Zimbabwe started in 1995 when canning companies imported the cultivar, Ex-Rico, which was locally called Michigan pea bean [7]. Before 1995, farmers in the Lowveld were producing navy beans mostly for subsistence since the production quantities were insignificant to supply to the industry due to lack of improved cultivars. By the year 2000, the production of Michigan pea bean had been widespread in the Lowveld region of Zimbabwe as a result of contract farming by canning companies [7]. However, cultivar release and commercialization of navy bean in Zimbabwe started in 2018 (Protea—released in July 2018, Caledona and Camellia—both released in December 2019) [7, 8]. Additionally, all the aforementioned navy bean cultivars were not bred for drought and heat tolerance despite their high grain yield potential under non-stressed environments. Moreover, some of the bean processors claim that the navy bean cultivar Protea sometimes clumps (grains stick together) after canning; as a result, the bean processing industry still imports, a process which increases production costs and affects the profitability of a canning operation. In addition, farmers also claim that Protea takes long to mature (110 days) despite being high yielding (yield potential of 4 t/ha) and tolerant to diseases of economic importance. Late-maturing genotypes require more irrigation cycles. This justifies the importance of participatory plant breeding approaches during cultivar development in order to develop cultivars that meet the needs and preferences of various actors along the bean value chain.

Participatory rural appraisal (PRA) relies on participation of the community (local people) and considers the value of stakeholders’ knowledge, skill, experience, their needs, preferences, abilities, and innovation [9]. Participatory rural appraisal has been extensively used to identify production constraints of many crops [10–17]. Ceccarelli et al. [18] and Morris and Bellon [19] reported that the participation of farmers’ in the initial breeding process provides insight into cultivar trait preferences, production and marketing constraints so that they can be addressed during the breeding process and hence enhance the adoption rate of newly developed cultivars. Information from PRA helps the breeder to design focussed breeding pipelines that result in genetic gains in farmers’ fields and more income to the farmers [20]. Mukankusi [21] and Ojwang et al. [22] successfully used information from PRA in the breeding process to develop common beans with resistance to fusarium root rot and bean stem maggot. According to our knowledge, there is no documented participatory research on navy bean production status, biotic stress management strategies, farmers’ perceived production and marketing constraints, and cultivar trait preferences among the major navy bean-growing regions in Zimbabwe. Although Katungi et al. [5] conducted a similar study in 2016 in Zimbabwe, the focus was not on the navy bean market class, but on common bean in general.

Furthermore, gender-sensitive breeding has been reported to improve adoption of released cultivars in Africa [16, 23–25]. It is, therefore, of paramount importance to consider the different roles and responsibilities of men and women when conducting PRA for the identification of farmers’ preferred traits, production and marketing constraints and biotic stress management practices. Danial et al. [20] reported that the adoption of new agricultural technologies such as improved cultivars is also affected by gender. Thus, the information from the baseline study will be used to develop an effective gender-responsive, demand-led, participatory plant breeding programme which considers the users’ views (needs and preferences). The results from the PRA will guide bean breeders in Zimbabwe in defining important traits and constraints, and in developing comprehensive breeding strategies to develop improved high yielding cultivars that are tolerant to biotic and abiotic stresses and preferred by value chain actors (consumers, traders, processors, and farmers). Therefore, the objectives of this study were to (i) identify major navy bean marketing and production constraints, (ii) identify navy bean cultivars and traits that are preferred by farmers, (iii) assess the production system of navy bean and (iv) identify the strategies used by farmers to manage drought and heat stress and their combined implications for breeding navy bean cultivars for Zimbabwe.

## Materials and methods

### Study area, sampling procedure and participants

The study was conducted in Chimanimani and Chipinge districts in the South East Lowveld region of Manicaland Province, Zimbabwe in November 2019. The area is a marginal semiarid region characterised by high temperatures and low, unpredictable and poorly distributed rainfall [13]. The two districts were selected based on prior information on their experience of growing navy beans [11] and on being the major navy bean-growing areas in Zimbabwe. In these areas, navy bean production occurs during the winter season (April to July) mainly under flood irrigation as a source of both food and income (Mubako, personal communication, 13 May, 2018^2^). Using a purposive sampling procedure [13, 26] to ensure good representativeness of navy bean-grower households in the study, a list of six major navy bean-growing villages namely Musikavanhu, Nenhowe, Nyanyadzi, Gudyanga, Maunganidze and Tonhorai located in the two districts were selected. These six villages were selected on the basis of their current high levels of navy bean production. Due to limited resources, the study could not be conducted in all the six villages. Therefore, out of these six villages which were used as the sampling frame, four [Nenhowe, Gudyanga, and Tonhorai (Chimanimani district) and Maunganidze (Chipinge district)] were chosen randomly for interviewing farmers, surveys and focus group discussions [26]. The Global Positioning System (GPS) location of the study areas, minimum and maximum temperatures, soil texture and mean rainfall totals are indicated in Table 1.

**Table 1:** Geographical description of the study locations

District	Village	Geographical location		Altitude (m.a.s.l)^**a**^	Mean annual rainfall (mm)^**b**^	Rainfall season	Soil types	Temperature (°C)^**c**^	
		Latitude	Longitude					Max^d^	Min^e^
Chimanimani	NW^f^	− 19.73976	32.43407	541	415	December–March	Sandy loamy	40	15
Chimanimani	GD^g^	− 19.89719	32.3819	491	450		Clay loamy	39	18
Chimanimani	TH^h^	− 19.93702	32.37236	492	430		Clay	40	16
Chipinge	MD^I^	− 19.95341	32.35402	499	120		Sandy loam	38	15

^a^*m.a.s.l* meters above the sea level

^b^*mm* millimetres

^**c**^***°****C* degrees Celsius

^d^*Max* maximum

^e^*Min* minimum

^f^*NW* Nenhowe

^g^*GD* Gudyanga

^h^*TH* Tonhorai

^I^*MD* Maunganidze

Source: Pambuka, personal communication, 11 November, 2019^1^; Matsenure, personal communication, 12 November, 2019^2^; Mukwakwami, personal communication, 13 November, 2019^3^; Masimura, personal communication, November 14, 2019^4^

^1^ Local agricultural extension worker for Gudyanga

^2^ Local agricultural extension worker for Nenhowe

^3^ Local agricultural extension worker for Maunganidze

^4^ Local agricultural extension worker for Tonhorai

For farmer surveys and focus group discussions, a systematic random sampling method was followed to identify navy bean farmers in the selected villages from lists provided by the local extension staff [13]. During the mobilization of farmers, which was done with the assistance of local agricultural extension workers, gender balance was considered accordingly to ensure that at least 50% of the participating farmers were females. A total of 176 (75 males and 101 females) navy bean-growing households were interviewed (Table 2).

**Table 2:** Number of farmers who participated in the individual household interviews and focus group discussions

Village	Household interviews			Focus group discussions		
	Males	Females	Total	Males	Females	No. of FGD^**a**^ conducted
Tonhorai	19 (37.3%)	32 (62.7%)	51	10	12	2
Maunganidze	22 (44.9%)	27 (55.1%)	49	10	10	2
Gudyanga	17 (34.7%)	32 (65.3%)	49	10	12	2
Nenhowe	17 (63%)	10 (37%)	27	10	11	2
Total	75 (42.6%)	101 (57.4%)	176	40	45	8

The values in parentheses indicate the percentage of male or female farmers who participated in the individual household interviews.

^a^*FGDs* focus group discussions

This was a representative sample of farmers who grow navy beans in the above-mentioned villages.

### Data collection and analysis

Data were collected using various PRA techniques which included transect walk, problem listing, ranking and analysis with key informants and corroborated by formal household interviews using a semi-structured questionnaire [13, 27]. Both formal and informal research approaches were used in the study in order to obtain high evidential value, to improve the precision, for validation, and to create a solid foundation for drawing conclusions [13]. The questionnaire had five components namely, demographic information, navy bean cropping systems (sole cropping, mono cropping, inter cropping and mixed cropping), farmers’ trait preferences of navy bean cultivars, navy bean production constraints and strategies used to mitigate some of these constraints. Mixed cropping is the production of two or more crops simultaneously on the same piece of land without row arrangements, whereas sole cropping is the growing of one crop cultivar in pure stands alone. On the other hand, inter cropping is the growing of two or more crops simultaneous on the same piece of land in alternate rows, whereas mono-cropping is the growing of a single crop on the same piece of land year after year. To eliminate gender dominance in discussions and gain an in-depth understanding of men and women farmer experiences in navy bean production and marketing, focus group discussions (FGDs) were conducted separately for men and women [13] with a group of community members (key informants, elders, women group representatives, community-based organization representatives, farmers, and village leaders) in each village. These farmers were selected based on their interest in the navy bean crop; they had grown navy beans in the last two consecutive years, knowledge of navy bean production, knowledge of the village history, and farmers’ influence in the village. In each of the four villages, two FGDs (one for men and one for women) were conducted. The number of participants in the FGDs in each village by gender is outlined in Table 2.

Issues discussed under the focus groups were navy bean farming systems, crop production practices, cropping calendar, preferred navy bean cultivars and reasons for preference within the community, ranking of production constraints, major diseases in order of importance, and heat and drought stress management strategies. Interviews were conducted in Shona, Ndau dialect, the local language with the help of enumerators that had been selected from these villages [11, 13]. Transect walks were conducted in three selected fields after the focus group sessions to promote discussion amongst farmers about the navy bean production systems and the associated constraints. The collected information was translated to English. The data that was obtained through FGDs, problem listing and transect walk was used for triangulation, to validate and support the data gathered from the individual semi-structured household questionnaire. The study was carried out in collaboration with the Ministry of Lands, Agriculture, Water, and Rural Resettlement.

Both qualitative and quantitative data from household interviews and FGDs were coded and subjected to analyses using cross-tabulation procedure, and contingency chi-square values were calculated for significant tests using the Statistical Package for Social Scientists (SPSS) (Release 21) computer package [28]. The data were classified as ordinal or nominal for the SPSS spreadsheet. For exploring frequencies and relationships, descriptive statistics such as percentage means, standard errors, minimum and maximum levels were computed for the data collected in each village. The farmers’ production and marketing constraints and trait preferences were ranked according to the frequency of citation by respondents (percentage of respondents who selected the respective constraint and trait) at village level and across the villages [15]. Charts were constructed in Microsoft Office Excel 2013.

## Results

### Demographics and household characteristics of respondents

As an economic activity, navy bean production is carried out by people of different ages in the villages sampled (Table 3). The age group 41–50 years had the highest number of respondents and accounted for 28% of the surveyed population (Table 3). There were also respondents above 70 years that were involved in navy bean production in all the areas surveyed.

**Table 3:** Distribution of respondents’ age in the study areas

		District				
Age interval (years)		Chimanimani villages		Chipinge village		
	Nenhowe	Gudyanga	Tonhorai	Maunganidze	Overall	***P*** value
< 30	1% (2)	3% (5)	3% (5)	1% (2)	8% (14)	0.294
31–40	2% (4)	10% (17)	5% (8)	3% (6)	20% (35)	0.001^*^
41–50	5% (8)	7% (13)	7% (12)	9% (16)	28% (49)	0.104
51–60	3% (6)	5% (9)	11% (19)	6% (10)	25% (44)	0.001^*^
61–70	2% (3)	2% (3)	3% (5)	6% (10)	12% (21)	0.098
> 70	2% (4)	1% (2)	1% (2)	3% (5)	7% (13)	0.429

The values in parentheses indicate the actual numbers per age group

^*^Denotes that the villages differed significantly at *P* ≤ 0.05

### Navy bean production, farming systems and important crops grown

Rainfall is unimodal, and seasons are classified accordingly. Summer season is characterized by rains from November to March/April and high temperatures, followed by winter season, which is dry and associated with low temperatures (April to July). The formal survey revealed that Maize (*Zea mays* L.), navy beans, onions (*Allium cepa* L.), wheat (*Triticum aestivum* L.), tomatoes (*Solanum lycopersicum* L.), velvet beans (*Mucuna pruriens* L.), sorghum (*Sorghum bicolor* L.), Lablab (*Lablab purpureus* L.) and okra (*Abelmoschus esculentus* L.) were the major food crops grown in the study areas (Table 4). Sunflower (*Helianthus annuus* L.), groundnut (*Arachis hypogaea* L.) and bambara groundnut (*Vigna subterranea* L.) were ranked as minor crops across all the villages. Most of the navy bean was grown during the dry winter season under irrigation using the scarce irrigation water resources alongside horticultural crops (onions and tomatoes) and wheat. Navy bean summer production was constrained by high temperatures and drought, even though some of the farmers grew navy beans in small plots for household consumption. The rest of the crops were mainly grown in summer. The food crops were ranked by both male and female farmers based on cultivation area, cash income and food security as outlined in the sections below.

**Table 4:** Important crops in terms of cultivation area, cash income and food security (percentage of respondents)

		Villages															
		Gudyanga (%)			Maunganidze (%)			Nenhowe (%)			Tonhorai (%)			Overall Mean			
								Sex									
Criterion	Crop	M^**a**^	F^**b**^	Mean	M	F	Mean	M	F	Mean	M	F	Mean	M	F	Mean	***P*** value
Cultivation area	Maize	48.8^1^	100^1^	74.4^1^	89.6^1^	65.2^1^	77.4^1^	100^1^	74^1^	87^1^	70.4^1^	100^1^	85.2^1^	77.2^1^	84.8^1^	81^1^	0.039^*^
	Beans	16.4^3^	57.2^2^	36.8^2^	32.8^2^	65.2^1^	49^2^	44.4^2^	29.6^2^	37^2^	39.2^2^	70.4^2^	54.8^2^	33.2^2^	55.6^2^	44.4^2^	0.044^*^
	Wheat	16.4^3^	16.4^4^	16.4^4^	0	0	0	29.6^3^	0	14.8^4^	8^4^	0	4^6^	13.5^6^	4.1^7^	8.8^6^	0.052
	Onions	24.4^2^	8.4^5^	16.4^4^	8^5^	40.8^3^	24.4^4^	14.8^5^	0	7.4^5^	8^4^	23.6^4^	15.8^4^	13.8^5^	18.2^4^	16^4^	0.050^*^
	Tomatoes	16.4^3^	48.8^3^	32.6^3^	32.8^2^	24.44^4^	28.6^3^	14.8^5^	0	7.4^6^	8^4^	39.2^3^	23.6^3^	18^3^	28.1^3^	23.1^3^	0.047^*^
	Sorghum	0	5.6^7^	2.8^7^	16.4^4^	8^5^	12.2^5^	29.6^3^	29.6^2^	29.6^3^	15.6^3^	8^5^	11.8^5^	15.4^4^	12.8^5^	14.1^5^	0.052
	Lablab	0	0	0	0	8^5^	4^6^	0	14.8^4^	7.4^6^	0	0	0	0	5.7^6^	2.9^8^	0.052
	Velvet beans	16.4^3^	8^6^	12.2^6^	0	8^5^	4^6^	0	0	0	0	0	0	4.1^7^	4^8^	4.1^7^	0.052
Cash Income	Maize	0	40.8^4^	20.4^4^	24.4^3^	65.2^1^	44.8^2^	44.4^2^	59.2^1^	51.8^2^	31.2^2^	70.4^1^	50.8^2^	25^2^	58.9^2^	42.0^2^	0.043^*^
	Beans	40.8^1^	89.6^1^	65.2^1^	40.8^1^	57.2^2^	49^1^	100^1^	29.6^2^	64.8^1^	54.8^1^	62.8^2^	58.8^1^	59.1^1^	59.8^1^	59.5^1^	0.044^*^
	Wheat	16.4^4^	8^6^	12.2^6^	24.4^3^	32.8^3^	28.6^3^	14.8^5^	14.8^3^	14.8^4^	8^5^	15.6^6^	11.8^5^	15.9^6^	17.8^5^	16.9^5^	0.051
	Onions	16.4^4^	48.8^3^	32.6^3^	24.4^3^	0	12.2^7^	29.6^3^	14.8^3^	22.2^3^	8^5^	8^7^	8^7^	19.6^4^	17.9^6^	18.8^4^	0.049^*^
	Tomatoes	32.8^2^	57.2^2^	45^2^	32.8^2^	24.4^4^	28.6^3^	0	14.8^3^	7.4^6^	31.2^2^	39.2^3^	35.2^3^	24.2^3^	33.9^3^	29.1^3^	0.047^*^
	Lablab	32.8^2^	0	16.4^5^	8^7^	8^7^	8^8^	29.6^3^	0	14.8^5^	0	23.6^5^	11.8^5^	17.6^5^	7.9^7^	12.8^6^	0.050
	Velvet beans	0	0	0	16.4^6^	16.4^5^	16.4^5^	14.8^5^	0	7.4^6^	15.6^4^	0	7.8^8^	11.7^7^	4.1^8^	7.9^8^	0.052
	Okra	0	16.4^5^	8.2^7^	8^7^	16.4^5^	12.2^6^	0	14.8^3^	7.4^6^	0	31.2^4^	15.6^4^	2^8^	19.7^4^	10.9^7^	0.050^*^
Food security	Maize	100^1^	100^1^	100^1^	100^1^	100^1^	100^1^	100^1^	100^1^	100^1^	100^1^	100^1^	100^1^	100^1^	100^1^	100^1^	NS
	Beans	8.0^2^	24^2^	16^2^	8.0^3^	16.4^2^	12.2^4^	14.8^2^	29.6^2^	22.2^2^	8.0^3^	23.6^3^	15.8^3^	11.6^3^	23.4^2^	17.5^2^	0.052
	Sorghum	0.0	0.0	0.0	16.4^2^	16.4^2^	16.4^2^	14.8^2^	14.8^3^	14.8^3^	15.6^2^	31.2^2^	23.4^2^	11.7^2^	15.6^3^	13.7^3^	0.051
	Wheat	0.0	8.0^3^	8.0^3^	16.4^2^	16.4^2^	16.4^2^	0.0	0.0	0.0	15.6^2^	8^4^	11.8^4^	8^3^	8.1^4^	8.1^4^	0.052

The values in parentheses indicate the percentage of respondents who selected the respective particular crop, and the superscript indicates the relative rank of the crop

^*^Denotes that the villages differed significantly at *P* ≤ 0.05

^a^*M* male

^b^*F* female

### Crops based on cultivation area

Maize (81%), navy bean (44.4%), tomatoes (23.1%), onions (16.3%) and sorghum (14.1%) were the major crops grown by both male and female farmers in the study areas in terms of cultivation area (Table 4). In each of the villages, maize was ranked first by more than 50% of both male and female respondents. In Nenhowe and Tonhorai villages, navy bean ranked second with 44.4% (males) and 29.6% (females), 39.2% (males) and 70.4% (females) of respondents, respectively, while at Maunganidze village, it was ranked equally with maize in importance (65.2%) among women farmers.

### Crops based on cash income

Based on cash income, and in order of ranking by the male and female farmers, navy bean, maize, tomatoes, onions and wheat were the major crops grown in the study areas (Table 4). In all the four villages, navy bean was ranked first by 49–65.2% of the respondents, while in Maunganidze, Nenhowe and Tonhorai villages, it ranked second after maize among female farmers only. In contrary to women, male farmers in all the villages ranked navy bean as the most important cash crop ahead of maize, tomatoes, onions and wheat. The ranking for the other crops is shown in Table 4.

### Crops based on food security

Regarding food security, maize (100% of respondents), navy beans (17.5%), sorghum (13.7%) and wheat (8.1%) were the major crops as indicated by both men and women (Table 4). In Gudyanga and Nenhowe villages, navy bean was ranked second by 8.0 and 14.8% of the male respondents, respectively, while in Maunganidze village among men, it occupied the third (8.0%) place after sorghum and wheat, which were ranked equally (16.4%). Sorghum (31.2%) occupied the second place at Tonhorai village among women, while navy bean occupied the same place at Nenhowe (29.6%) and Gudyanga villages among women.

### Land size and navy bean production yield

Sole cropping was the predominant (100%) cropping system in all the surveyed villages (Table 5). Farmers cultivated navy bean farming on small land holdings (mean = 0.27 ha). The average land size allocated to navy bean production was not significantly different among the villages ranging from 0.23 (Gudyanga) to 0.32 ha (Nenhowe) (Table 5). On average, the total land size per household was 0.75 ha, with the smallest being 0.69 ha (Gudyanga) and the largest being 0.77 ha (Tonhorai). The average grain yield of navy bean varied significantly (*p* < 0.05) from village to village with Gudyanga having the highest yields (Table 5). Focus group discussions reported average grain yields of 2.45, 2.76, 2.19 and 2.42 t ha^−1^ in Tonhorai, Gudyanga, Nenhowe and Maunganidze villages, respectively.

**Table 5:** Land size and navy bean cropping system in across four villages

	Average land size		Farming system (%)				Estimated yield (t ha^**-1**^)
	Average land size (ha) per household	Average land size (ha) allocated to navy bean (ha) per household	SC^**a**^	IC^b^	MC^c^	MOC^d^	
**Village**							
Tonhorai	0.77	0.27	100	0	0	0	2.45^bc^
Gudyanga	0.69	0.23	100	0	0	0	2.76^d^
Nenhowe	0.79	0.32	100	0	0	0	2.19^a^
Maunganidze	0.76	0.26	100	0	0	0	2.42^b^
Mean	0.75	0.27	100				2.45
LSD^e^	0.11	0.05	NS	-	-	-	0.26
*F* pr^f^	0.076^NS^	0.092^NS^	NS	-	-	-	0.035^*^

The % indicates the percentage of respondents who are using the respective farming system

Means followed by the same letter are not significantly different

*NS* non-significant

^*^P < 0.05

^a^*SC* sole cropping

^b^*IC* inter cropping

^c^*MC* mixed cropping

^d^*MOC* mono cropping

^e^*LSD* least significant differences of means (5% level*)*

^f^*F pr* probability value (5% level)

### Navy bean production constraints

Navy bean production was hampered by many constraints. Challenges ranged from biotic, abiotic and socio-economic constraints (Table 6). The perception of the constraints affecting navy bean production in the study locations was not different within and across villages as well as between men and women within the villages. The ranking of the constraints among both male and female farmers across all the locations did not differ much. Drought stress, heat stress, power outages/electricity cuts, susceptibility to pod shattering, poor soil fertility, insect pests, seed availability and diseases were the main constraints of navy bean production across all the villages according to both male and female farmers.

**Table 6:** Navy bean production constraints experienced by farmers

		Villages										
Sex	Constraint	Gudyanga (%) rank		Maunganidze (%) rank		Nenhowe (%) rank		Tonhorai (%) rank		Mean (%)	Overall rank	***P*** value
**Females**	Heat stress	60	2	64	2	48	2	60	2	58	2	0.046^*^
	Drought stress	84	1	80	1	100	1	80	1	86	1	0.025^*^
	Susceptibility to pod shattering	32	5	32	4	28	5	36	4	32	4	0.208
	Poor soil fertility	36	4	24	6	40	3	28	7	32	4	0.042^*^
	Diseases	24	7	24	6	28	5	28	7	26	8	0.417
	Insect pests	28	6	32	4	28	5	32	5	30	6	0.417
	Seed availability	24	7	24	6	28	5	32	5	27	7	0.152
	Low-yielding cultivars	20	10	20	10	20	10	20	9	20	10	0.591
	Power outages	48	3	52	3	44	4	40	3	46	3	0.083
	Shortage of labour	20	10	20	10	20	10	16	11	19	11	0.556
	Lack of access to transport	24	7	24	6	24	9	20	9	23	9	0.556
**Males**	Heat stress	48	3	44	2	60	1	68	1	55	2	0.018^*^
	Drought stress	76	1	84	1	68	2	64	2	73	1	0.028^*^
	Susceptibility to pod shattering	44	4	44	2	36	4	44	4	42	4	0.139
	Poor soil fertility	28	5	40	5	36	4	28	7	33	6	0.062
	Diseases	24	7	24	7	28	6	32	6	27	9	0.152
	Insect pests	28	5	28	6	28	6	36	5	30	8	0.139
	Seed availability	24	7	24	7	24	8	20	8	23	10	0.556
	Low-yielding cultivars	20	10	20	10	20	9	16	10	19	11	0.556
	Power outages	60	2	44	2	14	3	14	3	54	3	0.046^*^
	Shortage of labour	80	10	20	10	16	11	16	10	33	6	0.417
	Lack of access to transport	96	7	24	7	20	9	20	8	40	5	0.417

The % indicates the percentage of respondent; ^*^ denotes that the villages differed significantly at *P* ≤0

The most challenging insect pests across all the locations were the black bean aphid, bean stem maggot and harvester termites [*Hodotermes mossambicus* (Hagen) (Isoptera: Hodotermitidae)]. Diseases mainly comprised of bean rust (*Uromyces appendiculatus*), angular leaf spot (*Pseudocercospora griseola*), and common bacterial blight (*Xanthomonas axonopodis* pv. *phaseoli*). Drought stress was the second most challenging constraint in Nenhowe (68%) and Tonhorai (64%) villages among male farmers, while heat stress ranked the same at Gudyanga (60%), Maunganidze (64%), Nenhowe (48%) and Tonhorai (60%) villages among female farmers. Farmers reported that drought stress mainly occurred during the reproductive stage of growth, and heat stress was common in the late planted crop for a short period of time. The other major constraints reported by both male and female farmers were lack of access to transport, low yielding cultivars and shortage of labour.

### Management strategies for moisture and heat stress

The strategies used by farmers to alleviate the effects of moisture stress are summarized in Table 7. A total of 40, 38, 33 and 43% of farmers in Gudyanga, Maunganidze, Nenhowe and Tonhorai villages, respectively, did not use any strategy to manage/control moisture stress. However, soil mulching, reduced acreage, use of ridges, cultivating to retain more soil moisture, adjusting planting dates, and watering of plants at night are the strategies that were used by the other farmers to alleviate the effects of moisture stress. Soil mulching was the most widely used method of managing moisture stress at Gudyanga (29% of respondents), Maunganidze (16%) and Tonhorai (14%). During FGDs, farmers in all the four villages highlighted the importance of soil mulching in suppressing and reducing weed infestation and fungal disease pressure. At Nenhowe village, the most common method was the use of ridges (22%) followed by cultivating to retain more soil moisture. Overall (18%), soil mulching was the most common method of managing moisture stress as reported by 29, 16, 14 and 6% of farmers interviewed at Gudyanga, Maunganidze, Tonhorai and Nenhowe villages, respectively. Ridges (12%) were the second most widely used strategy, followed by reducing acreage (11%) and cultivating to retain more soil moisture (11%). Less common strategies of managing moisture stress included adjusting planting dates (3%) and watering of plants at night (6%).

**Table 7:** Management strategies for moisture and heat stress across four villages

		Village				Average (%)	
Stress	Strategy	Gudyanga (%)	Maunganidze (%)	Nenhowe (%)	Tonhorai (%)		***P*** value
**Moisture**	Soil mulching	29 (2)	16 (2)	6 (7)	14 (2)	18 (1)	0.040^*^
	Reducing acreage	11 (3)	5 (6)	17 (3)	14 (2)	11 (3)	0.051
	Use of ridges	9 (5)	14 (3)	22 (2)	9 (5)	12 (2)	0.049^*^
	Cultivating to retain more moisture in soil	6 (5)	14 (3)	11 (4)	14 (2)	11 (3)	0.053
	Adjusting planting dates	0 (7)	8 (5)	0 (7)	3 (6)	3 (6)	0.053
	No control strategy	40 (1)	38 (1)	33 (1)	43 (1)	39 (NC)^a^	0.052
	Watering of plants at night	6 (6)	5 (6)	11 (4)	3 (6)	6 (5)	0.053
**Heat**	Adjusting planting dates	18 (4)	22 (2)	56 (1)	38 (1)	29 (2)	0.006^*^
	Irrigating at night	24 (2)	49 (1)	25 (2)	21 (3)	32 (1)	0.027^*^
	No control strategy	24 (2)	16 (3)	6 (4)	25 (2)	19 (NC)	0.042^*^
	Mulching	33 (1)	14 (4)	13 (3)	17 (4)	20 (3)	0.041^*^

The % indicates the percentage of respondents using that respective strategy

^*^Denotes that the village differed significantly at *P* ≤ 0.05

^a^*NC* not a control/management strategy (represents percentage of farmers who reported that they do not use any control or management strategy)

The strategies used by farmers to alleviate the effects of heat stress are summarized in Table. 7. Irrigating at night (reported by 32% of respondents), adjusting planting dates (29%), and mulching (20%) are the methods that were used by farmers to alleviate the effects of heat stress. However, overall, 19% of the farmers across all the villages did not use any heat stress management/control strategy. A total of 56, 38, 22 and 18% of farmers at Nenhowe, Tonhorai, Maunganidze and Gudyanga villages, respectively, confirmed using the strategy of adjusting planting dates to alleviate the effects of heat stress. At Gudyanga and Maunganidze, the most commonly used methods were mulching (33%) and irrigating at night (49%), respectively. Overall (32%), irrigating at night was the most common method of alleviating the effects of heat stress as reported by 49, 25, 24 and 21% of farmers interviewed at Maunganidze, Nenhowe, Gudyanga and Tonhorai villages, respectively.

### Navy bean marketing constraints

Navy bean production was hampered by many constraints among which are lack of diversity in terms of buyers/off-takers, non-payment for produce in hard currency, delayed payment by contractor, low grain producer price and inflation eroding the value of the produce (Table 8). Non-payment for produce in hard currency was the top most challenging constraint among both male and female farmers at Gudyanga, Nenhowe and Tonhorai, while lack of diversity in terms of buyers/off-takers (92% of male respondents) and low grain producer price (100% of female respondents) were ranked the same at Maunganidze. The other major challenging constraints among both male and female farmers were lack of transport to ferry produce, non-transparent grading, expensive packaging material and breach of contract by contractor.

**Table 8:** Navy bean marketing constraints experienced by farmers

		Village						
Sex	Constraint	Gudyanga (%)	Maunganidze (%)	Nenhowe (%)	Tonhorai (%)	Mean (%)	Rank	***P*** value
**Male**	Delayed payment by contractor	72	60	24	68	56	3	0.033^*^
	Breach of contract by contractor	0	0	0	64	16	7	0.022^*^
	Low grain producer price	36	28	96	44	51	4	0.019^*^
	Lack of diversity in terms of buyers/off-takers	48	92	84	64	72	2	0.081
	None	0	0	0	20	5	NC^a^	0.107
	Expensive packaging material	48	20	0	0	17	7	0.021^*^
	Non-payment for produce in hard currency	100	52	100	96	87	1	0.062
	Inflation eroding the value of the produce	48	56	48	44	49	5	0.421
	Lack of transport to ferry produce	24	72	12	0	27	6	0.033^*^
	Non-transparent grain grading	24	20	24	0	17	7	0.081
**Female**	Delayed payment by contractor	72	64	20	56	53	3	0.054
	Breach of contract by contractor	24	28	0	44	24	6	0.065
	Low grain producer price	64	100	60	36	65	2	0.050^*^
	Lack of diversity in terms of buyers/off-takers	64	28	40	76	52	4	0.056
	None	0	0	30	24	13.5	NC	0.003^*^
	Expensive packaging material	28	0	40	0	17	7	0.028^*^
	Non-payment for produce in hard currency	100	40	100	100	85	1	0.148
	Inflation eroding the value of the produce	0	44	80	24	37	5	0.009^*^
	Lack of transport to ferry produce	24	28	0	0	13	9	0.157
	Non-transparent grain grading	0	48	0	12	15	8	0.025^*^

The % indicates the percentage of respondents experiencing the respective constraint

^*^Denotes that the villages differed significantly at *P* ≤ 0.05

^a^*NC* not a constraint (represents percentage of farmers who reported that they do not experience any marketing constraint)

### Farmer preferred traits for improvement during navy bean breeding

Across all the locations, farmers concurred that there was need for improvement of certain traits in the current cultivars during breeding based on their needs and preferences. The farmer-preferred traits for improvement are summarized in Table 9. Tolerance to heat (72% of the respondents), and drought (72%), resistance to diseases (72%) and insect pests (71%), maturity period (71%), grain yield (71%), pod size (69%), grain size (68%) and resistance to pod shattering (68%) were identified as the most important traits that needed enhancement by both male and female farmers across all the locations. Canning quality (26%) and nutritional value (iron and zinc) (28%) were the least important traits for improvement among both male and female farmers across all the villages. Generally, no gender differences were observed for farmers’ trait preferences across the villages.

**Table 9:** Farmers’ trait preference of a navy bean cultivar by sex for improvement during breeding

	Village														
	Gudyanga (%)			Maunganidze (%)			Nenhowe (%)			Tonhorai (%)					
Cultivar characteristic	M^**a**^	F^**b**^	Mn^**c**^	M	F	Mn	M	F	Mn	M	F	Mn	Mean (%)	Overall rank	***P*** value
Heat stress tolerance	76 (1)	68 (6)	72 (2)	68 (8)	67 (9)	68 (9)	71 (1)	70 (1)	71 (1)	75 (1)	75 (2)	75 (2)	72	1	0.125
Drought stress tolerance	70 (3)	73 (1)	72 (2)	71 (1)	75 (1)	73 (1)	71 (1)	70 (1)	71 (1)	74 (3)	69 (8)	72 (3)	72	1	0.083
Disease tolerance	70 (3)	72 (3)	71 (4)	71 (1)	75 (1)	73 (1)	71 (1)	70 (1)	71 (1)	72 (4)	72 (4)	72 (3)	72	1	0.059
Insect pest tolerance	68 (5)	68 (6)	68 (6)	69 (6)	66 (10)	68 (9)	71 (1)	70 (1)	71 (1)	75 (1)	76 (1)	76 (1)	71	4	0.985
Canning quality	30 (16)	27 (17)	29 (16)	31 (16)	30 (16)	31 (16)	33 (16)	20 (17)	27 (17)	21 (16)	11 (17)	16 (17)	26	17	0.011^*^
Short cooking time	68 (5)	67 (8)	68 (6)	68 (8)	63 (11)	66 (11)	69 (7)	66 (7)	68 (7)	62 (11)	73 (3)	68 (10)	68	8	0.116
Grain yield	68 (5)	72 (3)	70 (5)	71 (1)	75 (1)	73 (1)	71 (1)	66 (7)	69 (6)	72 (4)	68 (9)	70 (5)	71	4	0.469
Maturity period	72 (2)	73 (1)	73 (1)	71 (1)	75 (1)	73 (1)	64 (11)	70 (1)	67 (9)	68 (7)	71 (6)	70 (5)	71	4	0.221
Nutritional value iron, zinc	22 (17)	34 (16)	28 (17)	29 (17)	19 (17)	24 (17)	21 (17)	59 (11)	40 (16)	21 (16)	19 (16)	20 (16)	28	16	0.012^*^
Growth habit	44 (15)	40 (15)	42 (15)	39 (15)	35 (15)	37 (15)	44 (15)	43 (13)	44 (15)	35 (15)	46 (15)	41 (15)	41	15	0.019^*^
Plant height	54 (12)	49 (12)	52 (12)	53 (12)	50 (12)	52 (12)	50 (12)	43 (13)	47 (12)	58 (12)	52 (12)	55 (12)	52	12	0.560
Resistance to pod shattering	68 (5)	65 (10)	67 (8)	68 (8)	71 (7)	70 (6)	67 (10)	63 (10)	65 (10)	66 (9)	72 (4)	69 (8)	68	8	0.200
Pod size	68 (5)	64 (11)	66 (9)	69 (6)	69 (8)	69 (8)	69 (7)	70 (1)	70 (5)	68 (7)	71 (6)	70 (5)	69	7	0.854
Grain size	62 (10)	69 (5)	66 (9)	71 (1)	75 (1)	73 (1)	69 (7)	66 (7)	68 (7)	66 (9)	65 (11)	66 (11)	68	8	0.714
Grain taste	46 (13)	46 (14)	46 (14)	44 (13)	42 (13)	43 (13)	46 (13)	47 (13)	47 (12)	48 (14)	47 (13)	48 (14)	46	14	0.025^*^
Storability	62 (10)	67 (8)	65 (11)	65 (11)	75 (1)	70 (6)	71 (1)	59 (11)	65 (10)	70 (6)	68 (9)	69 (8)	67	11	0.504
Ease of shelling	46 (13)	47 (13)	47 (13)	44 (13)	41 (14)	43 (13)	46 (13)	47 (13)	47 (12)	50 (13)	47 (13)	49 (13)	47	13	0.521

The numbers in parentheses () indicate the rank of the respective trait

^*^Denotes that the villages differed significantly at *P* ≤0.05

^a^*M* male

^b^*F* female

^c^*Mn* average

### Sources of seed supply and cultivar preferences by farmers

**Fig. 1:**
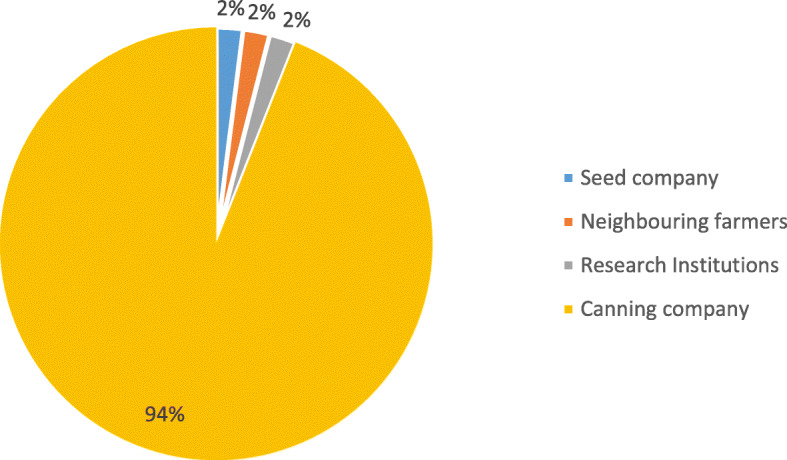
Sources of navy bean seed in South East Lowveld region of Zimbabwe in 2019

The major source of navy bean seed was the canning company (94%) as a business venture (Fig. 1). For household consumption, especially during summer season, the neighbouring farmers, research institutions and seed companies were a critical seed source. Most of the respondents were not well informed of the existence of improved navy bean cultivars such as Protea and Teabus. Zimbabwe White Bean formerly called Michigan pea bean was the most widely grown navy bean cultivar among both men and women farmers at Nenhowe (70% of respondents), Gudyanga (85%), Tonhorai (82%) and Maunganidze (90%) (Table 10). The second most widely cultivated navy bean cultivar was Teabus (Nenhowe—25%, Gudyanga—11%, Tonhorai—18% and Maunganidze—10%).

**Table 10:** Navy bean cultivars grown in the study areas

		Village										
Gender	Cultivar	Gudyanga		Maunganidze		Nenhowe		Tonhorai		Total	OveraIl rank	***P*** value
**Females**	Zimbabwe White Bean	54%	(1)	58%	(1)	15%	(1)	54%	(1)	49%	1	0.032^*^
	Teabus	8%	(2)	3%	(2)	10%	(2)	15%	(2)	9%	2	0.003^*^
	Caledon	3%	(3)	0%	(3)	0%	(3)	0%	(3)	1%	3	0.638
**Males**	Zimbabwe White Bean	31%	(1)	32%	(1)	55%	(1)	28%	(1)	34%	1	0.015^*^
	Teabus	3%	(2)	6%	(2)	15%	(2)	3%	(2)	5%	2	0.021^*^
	Caledon	3%	(3)	0%	(3)	5%	(3)	0%	(3)	1%	3	0.042^*^

The numbers in parenthesis () indicate the cultivar rank in terms of the number of respondents cultivating the cultivar

The % indicates the percentage of respondents who are growing the respective cultivar

^*^Denotes that the villages differed significantly at *P* ≤ 0.05

### Farmers’ desirable and undesirable characteristics of the navy bean cultivars

**Fig. 2:**
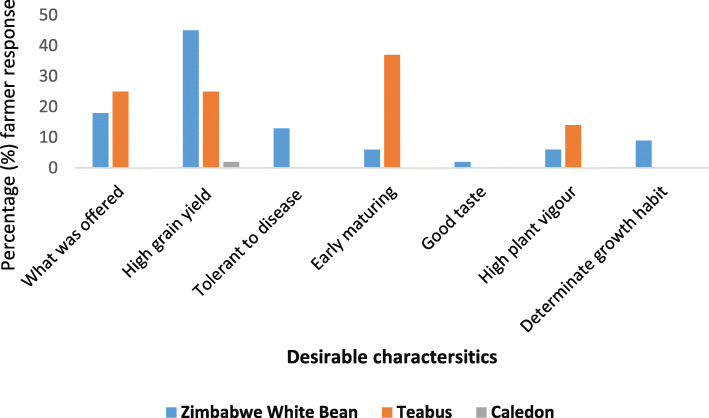
Desirable characteristics about the cultivars grown by farmers across the four villages

About 45 and 13% of the farmers across all the locations desired high grain yield and disease tolerance of the Zimbabwe White Bean cultivar, respectively (Fig. 2). However, more than 15% of the farmers indicated they did not like any attribute of the Zimbabwe White Bean and Teabus cultivars, but they were the only available cultivars that were offered by the contractor/canning company. Even though Caledon was not widely grown, 2% of the farmers who cultivated the cultivar liked the high grain yield potential of the cultivar.

**Fig. 3:**
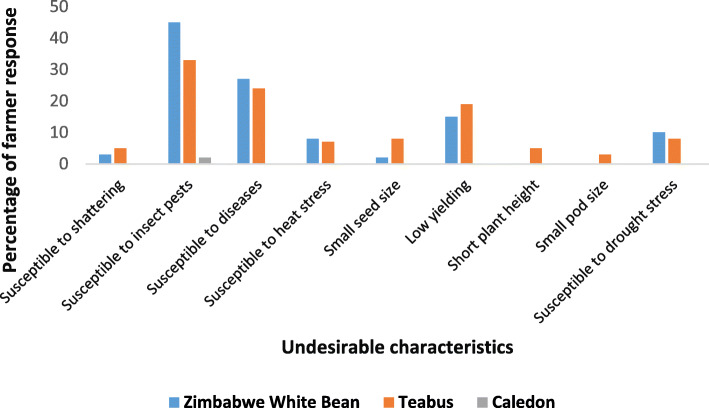
Undesirable characteristics about cultivars being grown by farmers across four villages

The undesirable characteristics of the navy bean cultivars grown by farmers across the four villages are presented in Fig. 3. Results indicated that susceptibility to pod shattering (3% of respondents), susceptibility to insect pests (45%), susceptibility to diseases (27%), susceptibility to heat stress (8%), small seed size (2%), low grain yield potential (15%) and susceptibility to drought stress (10%) were some of the undesirable traits of Zimbabwe White Bean cultivar. The cultivar Caledon had only one undesirable characteristic across all the locations, which was susceptibility to insect pests (2%), mainly the black bean aphid. With regards to Teabus, some of the undesirable characteristics across the four villages were susceptibility to pod shattering (5%), susceptibility to insect pests (33%), susceptibility to diseases (24%), susceptibility to heat stress (7%), small seed size (8%), low grain yield potential (19%), short plant height (5%), small pod size (3%) and susceptibility to drought stress (8%).

## Discussion

### Gender and age distribution of respondents

The majority of navy bean farmers were females except at Nenhowe where women cultivate green mealies as a cash crop since it fetches a high market price [29]. Traditionally, bean is considered a women’s crop in Zimbabwe. However, due to the profitability of navy bean production, men are slowly involved in the cultivation of the crop. A very small percentage of the farmers were aged below 30 years since most of the youths were engaged in diamond mining which is associated with quick cash returns. The socio-economic constraints associated with navy bean production also hindered the participation of youths in navy bean production. Njenga et al. [30] reported that agricultural activities were unattractive to youths due to the input and time investment.

### Important crops grown

Farming enterprises were business oriented evidenced by the type of crops grown which ranged from horticultural crops, cereals, and others. The diversification of crops protected farmers from natural hazards such as drought, guaranteeing food, nutrition and income security at farm level. Maize, navy bean and sorghum featured prominently among the most important food crops for household consumption and income generation. Both maize, sorghum and navy beans are complementary to each other within the farming households; navy bean is a cheap source of protein, and farmers often consume it with “sadza/isitshwala” (thick porridge made from maize/sorghum flour). Furthermore, navy bean is a short-season crop which allows relay-cropping with green mealies, and its market is guaranteed since it is grown under contract farming. Income generated from navy bean sales is used to purchase farming inputs and livestock (goats and cattle) and household food, and to pay school fees. Sorghum, a drought-tolerant crop was grown by farmers during the summer season under rain-fed conditions as a coping strategy for adapting to drought stress and guaranteeing both human food and animal feed. These findings agree with Chidoko and Zhou [31] and Nassary et al. [32] who reported maize and dry bean as important food and cash crops in many parts of the world, including Sub-Saharan Africa. Additionally, Katungi et al. [5] reported that maize and sorghum are the main food crops grown in natural region four (NR V) in Zimbabwe. The majority of men ranked navy bean (average of USD800 per tonne) as the most important crop for cash income ahead of maize (average of USD235 per tonne) which was ranked first by women. This was because men were in charge of navy bean sales with the contractor and women were in charge of green mealies (boiled, roasted) sales.

### Land size and navy bean production yield

The average land size of 0.27 ha per household allocated to navy bean production was comparable to the observation made by Katungi et al. [5] that the majority of dry bean farmers in Natural Region V allocated an average of 0.271 ha to dry bean production. Generally, navy bean was cultivated in small fields because most contracting companies availed seed which was enough to cover an area of 0.25 ha per farmer. This was used as a risk management strategy against biotic, abiotic and socio-economic constraints coupled with the unavailability of seed. Sole cropping was the most common cropping system because other crops (wheat, tomatoes and onions) which the farmers cultivate during the dry winter season are not compatible for intercropping with navy bean. Furthermore, the weather conditions during the winter season are not favourable for the cultivation of other main food crops which are compatible to intercrop with navy beans such as maize. This is corroborated by Katungi et al. [5] and Njoki [33] who found that dry bean was grown as a sole crop in Zimbabwe and Kiambu County of Kenya, respectively. However, some authors, Deressa et al. [14], Fageria et al. [34] and Mongi et al. [35] reported that dry bean was grown by smallholder farmers in intercropping and mixed cropping systems in other African countries. Gudyanga had the highest average navy bean grain yields (2.76 t ha^-1^) per hectare because farmers practiced good agricultural practices (GAPs) (optimum fertilizer application, pesticide application and herbicide application) despite the socio-economic constraints. In contrary, Nenhowe had the least average navy bean grain yields per hectare due to frequent breakdown of irrigation pumps which resulted in farmers applying an average of two irrigation cycles instead of five cycles per navy bean growing season. The significant yield gap between farmers’ yield and yield potential could be attributed to drought stress, heat stress, susceptibility to fungal diseases, and socio-economic constraints such as unavailability of seed of improved cultivars.

### Navy bean production constraints

The results revealed that drought stress, heat stress, pod shattering, poor soil fertility, insect pests, diseases and seed availability were the major production constraints. This is corroborated by Katungi et al. [5] who found that insect pests, diseases and drought stress are the most challenging dry bean production constraints in Zimbabwe. A similar study carried out by Chemining’wa et al. [36] in Kenya also reported that insect pests, diseases, drought and transport are challenging constraints of navy bean production. Many scientists [37–39] have reported that about 60 % of cultivated beans worldwide are grown under the risk of either terminal or intermittent drought. Drought stress and heat stress were the most important constraints because farmers are not able to grow navy beans during the main cropping season due to erratic rainfall totals (less than 450 mm) coupled with high temperatures (more than 32 °C) [40]. Load shedding (power cuts) in winter was also an important constraint because it affected the frequency of irrigation cycles resulting in the crop experiencing prolonged periods of moisture stress. Farmers were forced to irrigate at night when electricity was available, a strategy which was often a challenge to women farmers due to household responsibilities/duties. Tackling the effects of load shedding on irrigation cycles needs much more effort and a holistic approach; the government of Zimbabwe and private sector must invest in solar energy to minimize the effects of moisture stress when electricity is not available emanating from increased demand.

Susceptibility to pod shattering was an important constraint because despite experiencing significant grain yield losses, farmers spent a lot of time and labour in picking the small seeded grains from the ground. Farmers reported susceptibility to diseases as an important production constraint because some of the diseases infect the grain, and diseased grains are discarded by the contractor during grading since they do not meet the canning quality standards. This results in significant income losses on the part of the farmer since payment is based on the quantity of “clean” grain. Similar findings were reported by Njoki [33] and Mongi et al. [35] who found that diseases such as angular leaf spot were important dry bean production constraints in the southern highlands of Tanzania and Kiambu County in Kenya, respectively. The high susceptibility to diseases was exacerbated by the unavailability of seed of improved navy bean cultivars in the market. This was due to the absence of newly released cultivars and the lack of formal seed system (production of breeders, foundation and certified seed) for Zimbabwe White Bean to inject disease free seed into the system since this two-decade old cultivar was never formally released [6]. This results in continued recycling of infected seed, a scenario which increases the prevalence of seed borne diseases such as angular leaf spot. Similarly, Chemining’wa et al. [36] reported that navy bean seed systems in Kenya were informal. On the other hand, the South African cultivar “Teabus” was replaced in South Africa due to high susceptibility to fungal diseases such as bean rust [Fourie, personal communication, January 2020^3^]. The unavailability of improved navy bean seed in Zimbabwe is due to the fact that the first improved navy bean cultivar (Protea) in Zimbabwe was released in 2018 [6] such that seed companies are still bulking foundation and certified seed. This compels canning companies to rely on Zimbabwe White Bean and importation of Teabus seed from South Africa) increasing production costs). Consequently, the majority (94%) of farmers sourced their seed from canning companies due to the unavailability of seed in agro-dealer shops. This is corroborated by Chemming’wa et al. [36] who found that contracting companies in Kenya (Nakuru County) gave navy bean seed to farmers to grow the crop for them due to unavailability of seed. The challenge of the unavailability of navy bean seed and informal seed system can be partly addressed through the establishment of community-based seed production organizations (CBSPO). This will ensure that high quality seed of improved cultivars is available within the communities at affordable prices [41].

Poor soil fertility frequently appeared as an important constraint probably due to soil nutrient imbalances since most of the farmers applied basal and top-dressing fertilizers without conducting soil nutrient analysis. Similar findings were reported by Njoki [33] and Mongi et al. [35] who found that poor soil fertility was an important dry bean production constraint in the southern highlands of Tanzania and Kiambu County in Kenya respectively. This finding is also consistent with Vanlauwe et al. [42] who observed that unbalanced soil fertilization resulted in poor fertilizer response in maize. It is therefore of paramount importance for bean breeders to develop cultivars that are adapted to poor soils, particularly cultivars that are capable of producing acceptable grain yields under low nitrogen conditions.

### Farmer’s trait preferences

There was a fair level of consistency in trait preference (priority traits for improvement during breeding) rankings by both men and women probably because all the farmers experienced the same production and marketing constraints. This result is not in agreement with the findings of Asfaw et al. [24] who reported differences in trait preferences among both men and women dry bean farmers in Ethiopia. Tolerance to storage weevils, grain taste and cooking traits were not considered as important traits for improvement because farmers mostly grow navy beans for income generation, and also deliver most (90 %) of the produce to the contractor, and reserve 10 % for consumption. This prompts them not to value or put more weight on them. The high market value (USD 800 per tonne) of navy beans compared to other subsistence crops such as maize prompts farmers to reserve less for household consumption in search of income. These findings contradict with Katungi et al. [5], Sheikh et al. [17], Asfaw et al. [24], Njoki [33], and Balcha and Tigabu [43] who reported a significant number of farmers who preferred common bean cultivars with a good taste and short cooking duration in their studies. Farmers did not value the importance of invisible traits such as nutritional value (Fe and Zn) and canning quality probably due to the unfamiliarity with the nutritional and health benefits of consuming bio fortified cultivars in these two districts. Therefore, capacity building and bio fortified bean awareness creation campaigns should be intensified in these two districts to strengthen farmers’ knowledge on nutrition.

Generally, farmers showed strong preference for drought and heat stress tolerance ahead of high grain yield potential, suggesting that they are prepared to trade off a high yielding cultivar for a drought tolerant cultivar. This emphasizes that farmers perceived drought and heat stress as an urgent matter which the plant breeding program needs to address as a priority. Similar findings were reported by Derera et al. [13], who found that maize farmers in Mutare West of Zimbabwe selected the drought tolerance trait ahead of grain yield. These findings also concur with Asfaw et al. [24], Assefa et al. [25], Njoki [33], Assefa et al. [44], and Umar [45] who reported that farmers preferred drought tolerant dry bean genotypes for drought escape during their studies.

Farmers preferred early maturing cultivars for early household food security, drought escape and the reduction of the number of irrigation cycles due to insufficient water resources for irrigation. Early maturing cultivars can also be grown during the main short rainfall season in summer, and in winter, they escape heat stress and bean rust disease by maturing before temperatures begin to rise in July. Asfaw et al. [24], Balcha and Tigabu [43], and Assefa et al. [44] reported that dry bean farmers considered earliness as an important selection criterion in drought prone areas. Farmers preferred cultivars that are tolerant to pod shattering to reduce the amount of time and labour spent in picking the small seeded grains from the ground. This is corroborated by Asfaw et al. [24] who reported that farmers in Ethiopia preferred dry bean genotypes that were tolerant to pod shattering. Tolerance to diseases was one of the most preferred traits due to high costs of fungicides and the need to reduce the amount of labour and time spent on processing diseased grain, which was often done by women.

High grain yield was an important trait that was preferred by farmers because navy beans are mainly grown under contract farming, and high productivity usually translates to high income. These findings agree with Asfaw et al. [24], Balcha and Tigabu [43], and Assefa et al. [44], who reported that high grain yield was an important selection trait of dry bean farmers in Ethiopia. Farmers preferred cultivars with an indeterminate growth habit because they had more than one flash of flowering periods which increased chances of moisture stress escape.

### Cultivars grown by farmers

There was a narrow variability in terms of navy bean cultivars being grown by farmers partially due to the unavailability of seed of improved navy bean cultivars in the market. The majority of farmers predominantly grow Zimbabwe White Bean since the year 2000 because this is what was being offered by canning companies, but reckoned that it was not the ideal cultivar due to a number of undesirable characteristics. Teabus was grown by a small fraction of farmers due to the limited quantities of seed available emanating from the high costs associated with the importation of seed from South Africa. On the other hand, the cultivar Caledona was only grown by very few individuals at Gudyanga and Nenhowe because the cultivar had not been officially released into the market. The private seed company Seed Co released Caledona in December 2019, therefore farmers who grew Caledona might have obtained the seed through on-farm variety pre-release demonstration plots.

### Management strategies for moisture and heat stress

Soil mulching with maize stover was widely used as a moisture and heat stress management strategy to conserve soil moisture and regulate soil temperature respectively due to its multi beneficial effects. As reported by Iqbal et al. [46], Kader et al. [47], and Telkar et al. [48], soil mulching improves the water infiltration and retention capacity of the soil, and also reduces surface evaporation resulting in higher water use efficiency. Many scientists (Iqbal et al. [46], Kader et al. [47], Telkar et al. [48], Bodner et al. [49], Lamont [50], and Long et al. [51]) report that mulches protect the soils from extreme temperatures by lowering soil surface temperatures thereby keeping the plant root zone cooler and preventing soil temperature fluctuations, which is beneficial for overall crop growth. Farmers revealed that soil mulching had many other beneficial effects which included a reduction in the incidence of fungal diseases and weed infestation.

Despite the wide use of soil mulching, it attracted harvester termites which reduced plant stand in navy bean by feeding on the plant. This agrees with Long et al. [51] and Nyagumbo et al. [52], who reported that the application of crop residues or organic mulches increases the activity of termites as a result of the moist conditions in the underlying soil. This suggests that breeding for tolerance to moisture stress must be a priority in navy bean breeding programs. Irrigating at night was an important moisture and heat stress management practice because it resulted in more water penetrating the soil due to the reduction in water losses from evaporation. However, female farmers found it difficult to irrigate at night due to the cultural household roles and responsibilities such as cooking. The strategy of irrigating at night agrees with Mahmoud and El-Bably [53] who reported that night time irrigation improved water productivity by reducing losses of evaporation. Dong et al. [54] reported that the application of irrigation water at night reduced the root-zone soil temperature by 0.6 °C in maize resulting in improved plant growth.

Farmers frequently cultivated their fields with hoes as a drought stress management strategy to retain more soil moisture since more water penetrates into the soil instead of running away over the soil surface during irrigation. However, they highlighted that this method was very laborious. This is in agreement with Leslie [55] who reported that cultivation can be employed to retain more soil moisture bank levels for use by the crop. Farmers reduced the acreage under navy beans as a moisture management strategy because this shortened the irrigation cycle turn-over despite its negative implications on the overall production (output). The farmers often adjusted the planting dates (early planting in March) to avoid high temperature stress during the reproductive stages of development in July and August based on weather forecast information obtained from agricultural extension officers. This is corroborated by Akter and Islam [56], Asseng et al. [57], Chapman et al. [58], and Sandhu et al. [59] who reported that the effects of heat stress can be managed through the adjustment of relevant agronomic practices such as the adjustment of planting dates.

### Navy bean marketing constraints

Farmers revealed that most of the navy bean contractors were paying an average of USD800 per tonne, a price which they feel was low compared to what middlemen were paying (average of USD1200) for the sugar bean grain market class. This could be due to lack of diversity in terms of buyers/off-takers in the navy bean market. It was mainly the canning companies who were contracting farmers to produce navy bean grain and subsequently purchasing the grain from them such that there was no competition from other players/off-takers/middlemen. Most of the canning companies only purchase the navy bean grain if the source of seed is from them since the grain is meant for canning purposes such that canning quality is very important. These findings agree with Chemining et al. [36] who found that common bean farm gate prices were higher than the prices of navy bean in Kenya. Additionally, some of the contractors would frequently breach the signed contract by deviating from the agreed buying price per tonne in preference of a low buying price during grain collection time. Moreover, some of the farmers produced navy bean grain under contract farming without signing any contract, making it difficult to tackle disputes. This gave room to the contractor to manipulate the price of the inputs (seed) that would have been advanced to the farmer under contract farming and the buying price to his/her own advantage.

Farmers mentioned that the breach of contract and low grain producer price forced some of the farmers to withdraw from contractual agreements with canning companies in preference of bean seed production, also under contract with various seed companies. Similarly, Chemining’wa et al. [36] reported that the navy bean farmer–processor contractual agreements collapsed in Kenya in 1994 due to low grain producer prices that could not cover production costs. The constraint of low grain producer price was exacerbated by the delayed payment for the grain by the contractor coupled with inflation. Farmers highlighted that by the time the contractor processed their payments, the money would have lost value due to inflation thus negatively affecting their purchasing power. Delayed payment for the produce meant that most of the farmers did not have enough running capital to purchase inputs for the summer cropping season. Due to the acute shortage of hard currency (local Zimbabwean dollar) [60] in Zimbabwe, most of the contractors have not been able to pay for the produce in hard currency. Payments to navy bean farmers are made through bank transfers in form of Real Time Gross Transfer (RTGS). However, most of the farmers in the study villages do not own bank accounts such that they receive their payments through mobile money platforms such as EcoCash, Telecash and One Money. Some of the EcoCash agents are charging excessive premiums [61] above the authorized commission levels, up to 55% [62] of the mobile money being cashed out, eroding the farmers’ earnings.

### Farmers’ desirable and undesirable characteristics of the navy bean cultivars

Farmers highlighted that Zimbabwe White Bean was only tolerant to diseases when planted early, meaning that the cultivar was not tolerant but “escaped” disease infection. The early maturity trait of Zimbabwe White Bean ensured early returns and cropping fit since the farmers produced green mealies after harvesting navy beans. On the other hand, some of the farmers did not like any attribute on Zimbabwe White Bean and Teabus but they are cultivating the cultivars due to lack of options to select suitable cultivars for production. Caledona was preferred because of its tolerance to pod shattering, a trait which reduced labour in picking the grains from the ground and grain losses due to shattering.

Farmers highlighted that both Teabus and Zimbabwe White Bean were highly susceptible to drought and heat stress because both cultivars were not bred for production under heat-stressed and moisture-stressed environments. Therefore, there is need to disseminate improved navy bean cultivars in the Lowveld region that are tolerant to multiple biotic and abiotic constraints (drought, heat, and fungal diseases). In addition, farmers did not like the small seed size (< 25 g per 100 seeds) of Zimbabwe White Bean and Teabus because it presented challenges during grading (removal of chuff). Furthermore, the small-seeded navy been cultivars require more grain to fill a 50-kg bag compared with the large-seeded cultivars (> 40 g per 100 seeds) which required less. Unfortunately, most of the navy bean cultivars with good canning qualities are small seeded, and seed size (< 25 g per 100 seeds) is an important quality parameter for consideration during canning quality analysis due to consumer preferences [63].

## Conclusions

Culinary characteristics were not considered important traits for improvement during breeding since navy bean production is strictly a business venture. The main marketing constraints were non-payment for produce in hard currency, lack of diversity in terms of off-takers, delayed payment by the contractor, and low grain producer price. Farmers identified drought stress, heat stress, diseases, insect pests, unavailability of seed of improved cultivars, susceptibility to pod shattering and poor soil fertility as the major navy bean production constraints. Drought tolerance, heat tolerance, disease tolerance, insect pest tolerance, grain yield, resistance to pod shattering and early maturity were the major farmer-preferred traits. Improving seed size, pod shattering tolerance, fungal disease tolerance, drought tolerance and heat tolerance of the cultivar “Zimbabwe White Bean” predominantly grown in the Lowveld region without compromising on its short maturity duration, short cooking time, and sweet taste would potentially have a large impact on farmers’ livelihoods in the study areas. Navy bean was frequently grown under contract farming across the studied locations reducing the burden of sourcing inputs on the part of the farmer.

## Recommendations

These findings imply that plant breeders should employ participatory plant-breeding strategies and conventional approaches to improve existing cultivars and also develop improved climate smart cultivars. Therefore, navy bean improvement programs should consider and integrate the farmer-preferred traits, marketing, and production constraints during the development of improved cultivars. There is urgent need to hasten seed multiplication, dissemination of improved navy bean cultivars and extension services in awareness creation among farmers about improved navy bean cultivars in the Lowveld region. This is important considering that most of the respondents were not well informed of the existence of improved navy bean cultivars such as Protea and Teabus despite the fact that participatory variety selection was conducted in the Lowveld region. There is also a need for breeders to develop cultivars that are adapted to low soil fertility, particularly cultivars that are capable of producing acceptable grain yield under low nitrogen conditions. Breeding programs should also consider traders and processors trait preferences during cultivar development. Where navy bean breeding cannot incorporate all the preferred traits, the key attributes should be included in particular cultivars, making sure that maturity duration is short and there is no grain yield penalty since both are essential traits for farmers. Early maturing cultivars (i.e. less than 75 days) with tolerance to drought and heat stress are recommended for deployment in the very dry and hot areas such as the Lowveld region. Since navy bean is mainly utilized in the canning industry, the product profile should have cooking characteristics that meet industry demand. Agronomic practices such as irrigating at night, mulching, ridges, reduced acreage, and cultivating the soil to increase the water infiltration rate must be adopted by farmers to mitigate the effects of biotic and abiotic stresses. There is need to train navy bean farmers on contract farming, and CBSPO should be established to increase the availability of seed. Lastly, community seed banks should be established to improve access to seed reserves when varieties fail, protect knowledge related to diverse local varieties adapted to local conditions and reduce dependence on seed sources from outside the region. Irrigation schemes must be solarized to reduce the effects of power cuts on irrigation scheduling.

## Data Availability

The datasets used and/or analysed during the current study are available from the corresponding author on request.

## Abbreviations

FGDs: Focus group discussions
SSA: Sub-Saharan Africa
SE: South east
PRA: Participatory rural appraisal
GAPs: Good agricultural practices
CBSPO: Community-based seed production organization

## Footnotes

1 Head Research & Development at Cairns Foods Limited (One of the biggest navy bean canning companies)

2 Agricultural Extension Supervisor for Nenhowe, Gudyanga, Nyanyadzi, Chakohwa and Tonhorai villages

3 Dry Bean Breeder at Agricultural Research Council, South Africa
